# Transmission of SARS-CoV-2 during air travel: a descriptive and modelling study

**DOI:** 10.1080/07853890.2021.1973084

**Published:** 2021-08-31

**Authors:** Jinjun Zhang, Fei Qin, Xinyan Qin, Jianren Li, Sijia Tian, Jing Lou, Xuqin Kang, Huixin Lian, Shengmei Niu, Wenzhong Zhang, Yuguo Chen

**Affiliations:** a Beijing Emergency Medical Center, Beijing, China; b School of Electronic Electrical and Communication Engineering, University of Chinese Academy of Sciences, Beijing, China; c Uninted Family Healthcare, Beijing, China; d Emergency Department, Qilu Hospital, Shandong University, Jinan, China

**Keywords:** SARS-CoV-2, COVID-19, infectious diseases, transmission, aircraft, air travel

## Abstract

**Objectives:**

To explore the potential of SARS-CoV-2 spread during air travel and the risk of in-flight transmission.

**Methods:**

We enrolled all passengers and crew suspected of being infected with SARS-CoV-2, who bounded for Beijing on international flights. We specified the characteristics of all confirmed cases of COVID-19 infection and utilised Wells-Riley equation to estimate the infectivity of COVID-19 during air travel.

**Results:**

We screened 4492 passengers and crew with suspected COVID-19 infection, verified 161 confirmed cases (mean age 28.6 years), and traced two confirmed cases who may have been infected in the aircraft. The estimated infectivity was 375 quanta/h (range 274–476), while the effective infectivity was only 4 quanta/h (range 2–5). The risk of per-person infection during a 13 h air travel in economy class was 0.56‰ (95% CI 0.41‰–0.72‰).

**Conclusion:**

We found that the universal use of face masks on the flight, together with the plane's ventilation system, significantly decreased the infectivity of COVID-19.

**KEY MESSAGES**
- The COVID-19 pandemic is changing the lifestyle in the world, especially air travel which has the potential to spread SARS-CoV-2.
- The universal use of face masks on the flight, together with the plane's ventilation system, significantly decreased the infectivity of COVID-19 on an aircraft.
- Our findings suggest that the risk of infection in aircraft was negligible.

## Introduction

The SARS-CoV-2 pandemic has caused unprecedented public health problems and economic disruption, especially travel [1–3]. Given the serious risk of importation of COVID-19 cases during the pandemic, many countries have abated or cancelled outbound flights according to the International Air Transport Association (IATA). By the first quarter of 2020, global air travel has slumped by 70 percent and European flights have dropped by 90 percent [4–6]. The Chinese government suspended outbound flights from other countries in March, and all international flights bound for Beijing have been diverted to 12 designated cities in mainland China since 23 March 2020 [7]. With the development of globalisation and the convenience of international air transportation, delineation of the characteristics and potential routes of transmission of SARS-CoV-2 in aircraft is expedient. Whether healthy passengers will be infected by patients in the adjacent seats and how SARS-CoV-2 spreads in aircraft should be addressed. Given that, we collected data on the passengers bound for Beijing to infer whether passengers might be infected, described the characteristics of the confirmed cases and estimated the infectivity of COVID-19 during air travel.

## Methods

### Study design and participants

This is a retrospective study. We enrolled passengers and crew suspected with COVID-19 infection who were bound for Beijing Capital International Airport (PEK) *via* international flights from 1 March to 31 March 2020. Passengers and crew were defined as a confirmed case with COVID-19 infection according to the new coronavirus pneumonia diagnosis and treatment program published by the National Health Commission of the People’s Republic of China [8]. The study was approved by Ethics Committee of Beijing Emergency Medical Centre (No.2020-01), and written informed consent was waived.

### Data sources and procedures

**Figure 1.**
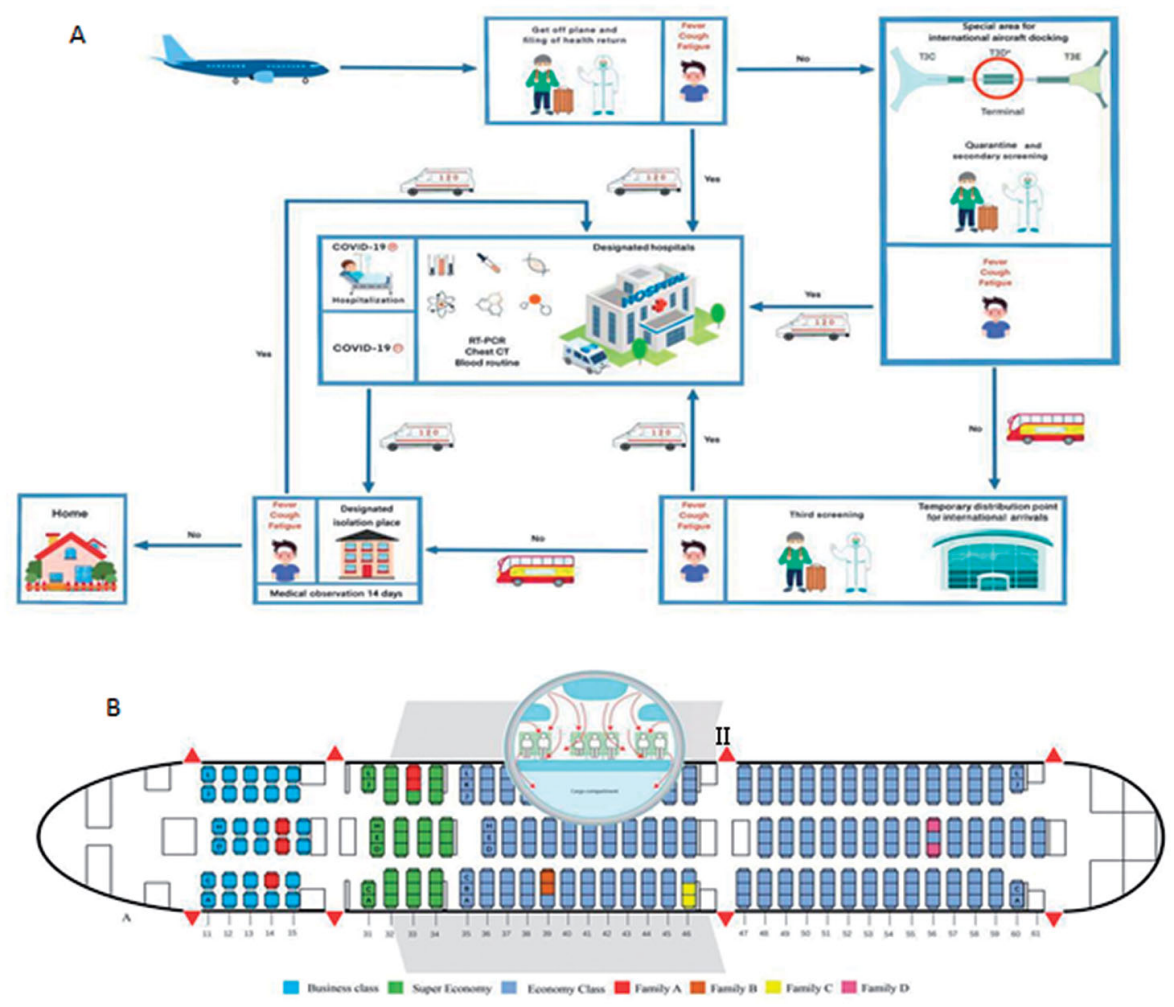
Screening flow and diagram of aircraft. (A) Screening flow; (B) Diagram of the Boeing 787-9 aircraft. I: Schematic diagram of seating plan with 11 confirmed cases; II: Air circulation pattern in passenger cabin, arrows show air currents from the Lancet^22^.

PEK is one of the largest airports in the world. To effectively prevent the outbreak of COVID-19, PEK designated T3 terminal D (T3-D) as a special area for international aircraft docking. All passengers on board reported the health on arrival. When a flight stopped at T3-D, all passengers underwent health quarantine inspection again, including body temperature screening, and health declaration. The healthy passengers were transferred to the designated temporary place of isolation for 14 days of medical observation, while others suspected of having COVID-19 infection were transferred to the designated hospitals where reverse-transcriptase polymerase-chain-reaction (RT-PCR), chest radiograph, and blood routine were performed (Figure 1(A)).

Data included demographic information, epidemiological characteristics, clinical data, laboratory test results for SARS-CoV-2, diagnostic test type, case clustering, and outcome. Data were collected by EMS providers in T3-D of PEK. Clinical outcomes were followed up until 1 August 2020. If data were missing from medical records, they were obtained by direct communication with EMS providers. The data of flights and passengers were obtained from the information desk and website of PEK.

### Statistical analysis

Continuous variables were expressed as means and standard deviations, and the categorical variables were presented as percentages in each category. All statistical analyses were performed with SPSS software version 22.0 (SPSS, Chicago, IL, USA).

The reproduction number (R0) has been widely utilised to evaluate the risk of large scale outbreak [5,9,10], but R0 is less capable to represent the infectivity risk in short time scale within a confined space, such as air travel last only a few hours. As reported, the quantitative microbial risk assessment method has been employed to model and predict the risk of infection for seasonal influenza, influenza A (H1N1) and Middle East respiratory syndrome coronavirus (MERS-CoV), where the infectivity is quantified with infectious quanta released by one source case per hour [11–13]. Quantum was defined as the necessary infectious droplets or aerosols to infect (1-*e*^−1^) exposed individuals. The analyses output were further processed with non-steady Wells-Riley equation to estimate the quanta release rate of COVID-19. We modelled the accumulation of infectious quanta with ordinary differential equation:

$$vS\frac{dN}{dt}=I\alpha q-NQ$$

where N is the concentration of quanta in the confined cabin, S is the number of susceptible in the cabin, v is the averaged space for each individual, I is the number of infectors, and q is the quantum generation rate. We assumed all passengers in the cabin being well equipped with face masks, which contributed to α as an absorption ratio on released quanta. We also assumed all the airline operators have maximised the filtering capability of ventilation system in the flight, which contributed to Q as an equalised air exchange rate with both filtering and replace effect. The above equation modelled the fact that the accumulation of quanta is equal to the generated quanta by infectors minus removed quanta by ventilation system. With simple algebra, the average number of quanta breathed by one susceptible can be derived:

$$\bar{\mu }=\beta \frac{I\alpha \mathrm{qpt}}{Q}[1-\frac{vS}{Qt}(1-e^{-\frac{Qt}{vS}})]$$

where p is the breathing rate, β is also contributed by face mask to decrease the breathed quanta. With the original definition of quantum, the probability of infection during the flight were modelled as:

$$P=\frac{D}{S}=1-e^{-\bar{\mu }}=1-e^{-\beta \frac{I\alpha \mathrm{qpt}}{Q}[1-\frac{vS}{Qt}(1-e^{-\frac{Qt}{vS}})]}$$

With the investigation results, the confirmed cases as well as their contact tracing were identified. The number of passengers and flight time were obtained through public source. We assumed an averaged space of 2 m^3^ for each passenger in the flight, an averaged breathing rate of 0.3 m^3^/h, an averaged air exchange ratio of 25 times per hour with 99.99% filtering efficiency, and the face mask efficiency to prevent quanta releasing and absorption of 90%. The quantum generation rate of COVID-19, i.e. q can then be estimated, which can be further utilised to predict infection risk in various scenarios. All estimations and predictions were performed with Matlab (version R2016).

## Results

### The risk of in-flight transmission during air travel

**Figure 2.**
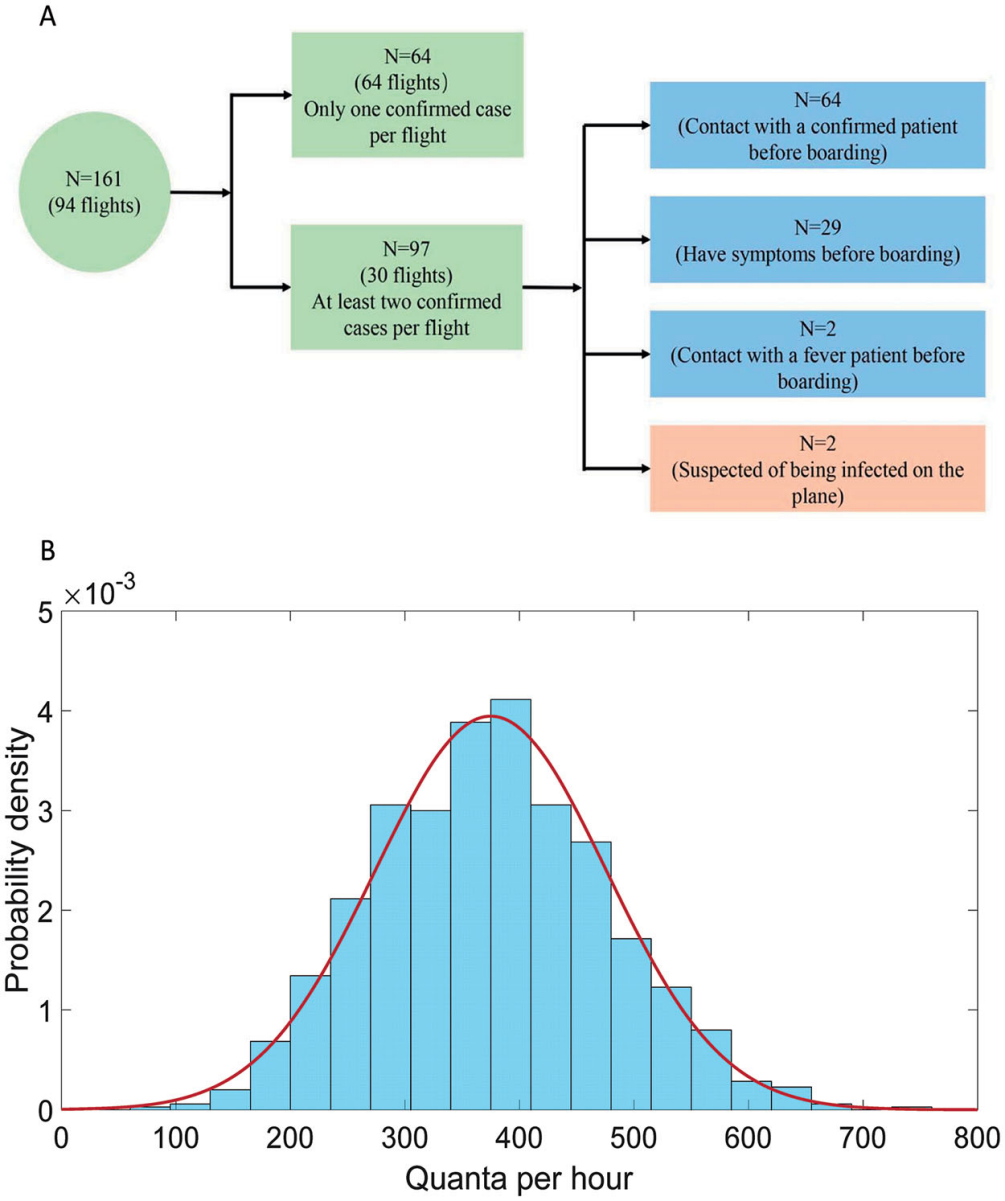
The inference process of the SARS-CoV-2 infectivity in aircraft. (A) The classification of 161 confirmed case; (B) The estimated rate of quanta generation in aircraft.

From 1 March to 31 March 2020, a total of 130 000 passengers arrived at PEK by more than 830 international flights, an average of 156 passengers per flight. Almost every passenger used a face mask to protect themselves, and some even wore medical protective clothing and goggles during air travel. In total, 4492 (3.4%) of 130 000 passengers were screened for symptoms of COVID-19 infection on arrival at PEK and isolated for 14 days of medical observation in the designated place of isolation; 161 (3.6%) passengers were verified as laboratory-confirmed cases of COVID-19 infection during quarantine (131, 81.4%) and medical observation (30, 18.6%) . Of 830 international flights, 94 (11.2%) carried confirmed cases. In these 94 flights, 64 (68.1%) flights had only one confirmed case and 30 (31.9%) flights had at least two. The overall number of confirmed cases in these 30 flights was 97, making a total of 161 confirmed cases. The number of confirmed cases in these 30 flights ranged from 2 to 11 per flight (Table 1). Specifically, eleven patients confirmed to have COVID-19 had close contact history and came from four families in the same aircraft, a Boeing 787-9 from Madrid to Beijing with flight duration of 10 h 18 min. However, no passengers seated within two rows of the confirmed cases were infected during air travel (Fig 1B). After investigation, sixty-four of 97 confirmed cases had ascertained epidemiological links by contact tracing before boarding, twenty-nine confirmed cases had symptoms before boarding, and two confirm cases had contact with fever patient before boarding. Only two (1.2%) confirmed cases were unable to determine where, when, and how they were infected, nor could they be excluded from being infected in the aircraft (Figure 2(A)).

**Table 1.** The top ten flights with confirmed and suspected infection cases.

Flight	Model of Aircraft	Duration of Flight	Number of passangers	Number of confirmed cases	Suspected infection case	AR
1	787-9	10 hr 18 min	247	11	0	0
2	747-8	8 hr 35 min	312	8	0	0
3	380–800	6 hr 53 min	369	5	0	0
4	777–300	5 hr 17 min	219	5	0	0
5	787-9	6 hr 51 min	294	4	0	0
6	787-9	10 hr 12 min	240	4	1	4.2‰
7	777–300	6 hr 32 min	256	4	0	0
8	330–300	10 hr 4 min	275	4	0	0
9	380–800	6 hr 44 min	352	4	0	0
10	777–300	8 hr 48 min	278	3	1	3.6‰

### Estimations and predictions by Wells-Riley equation

**Figure 3.**
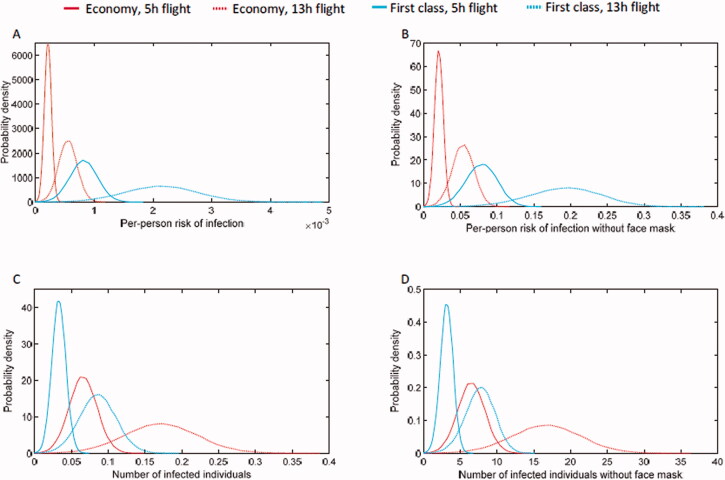
The predicated transmission of SARS-CoV-2 in aircraft. (A) The per-person risk of infection due to one infector. (B) The per-person risk of infection, assuming without face mask protection. (C) The predicted number of infected individuals due to one infector. (D) The predicted number of infected individuals, assuming without face mask protection.

We assumed these two confirmed cases as indeed infected during the air travel (D), the sixty-four confirmed cases with close contact as infectors during the air travel (I), and all other confirmed cases as randomisation following Bernoulli(0.5) distribution constrained by at least one infector in the flight. Figure 2(B) shows the estimated q with maxim likelihood estimation, about 375 quanta per hour (range 274–476). As a comparison, the infectivity of H1N1 was reported about 100 quanta per hour (range 79–128), while about 50 quanta per hour (range 6–140) for MERS-CoV. This demonstrated the significantly increased infectivity for COVID-19, while was less capable to character the per person risk during the flight. An intuitive variation of q, the effective infectivity can be defined with $q_{e}=\alpha \beta q,$ which was only 4 quanta per hour (range 2–5). The extremely low effective quanta will result in an expectation of per-person infection risk at only 0.56‰ (95% CI 0.41‰-0.72‰) during a 13 h air travel in economy class with one infector, or equalised 0.17 infected individuals. As a comparison, if all the passengers were not carefully protected with face masks, the number of infected individuals could be roughly 6 for a 5 h flight, and 17 for a 13 h flight in economy class. If the passengers travel in first class, larger space with less passengers, the number of infected individuals could be 3 for 5 h flight and 8 for 13 h flight. But the per-person risk for the passengers travel with first class would be higher given the same constraint of one infector in the cabin. In detail, the per-person risk during a 13 h flight in first class could be 2.2‰, which is about four times higher than travel in the economy class (Figure 3).

### Clinical characteristics of COVID-19 confirmed cases

Regarding the origin of the 161 confirmed cases, the foremost countries were England (53, 32.9%), Spain (46, 28.5%), Italy (18, 11.2%), United States (17, 10.5%), and France (7, 4.3%). Most patients (99, 61.5%) were female, the mean age was 28.6 years (range 1–70), and 53 (32.9%) patients were students. Sixty-five (40.4%) patients had direct exposure to a confirmed case, 77 (47.8%) had epidemiological contact history. 57 were associated with clustering involving at least two confirmed cases in a family or other places of close contact within 14 days, and 40 (24.8%) had familial clustering. Forty (24.8%) patients were asymptomatic during the flights while 121 (75.2%) had symptoms of respiratory infection, the most common symptoms were cough (34.8%), fever (32.9%), and fatigue (14.3%). The mean time from arrival to illness onset was 2.4 days, and from arrival to confirmed infection 2.7 days. By August 1, 2020, all 161 patients had been discharged, no died (Table 2).

**Table 2.** Characteristics of 161 patients with COVID-19 infection.

	Case (*n* = 161)
Sex, *n* (%)	
Female	99 (61.5)
Male	62 (38.5)
Age, years	
Mean (SD)	28.6 (12.8)
Range	1–70
Occupation, *n* (%)	
Crew	4 (2.5)
Student	53 (32.9)
Employee	29 (18.0)
Other	75 (46.6)
Cluster case, *n* (%)	57 (35.4)
Family	40 (24.8)
Other	17 (10.6)
Contact history, *n* (%)	77 (47.8)
Contact with confirmed case	65 (40.4)
Contact with suspected case	12 (7.5)
Asymptomatic, *n* (%)	40 (24.8)
Signs and Symptoms, *n* (%)	121 (75.2)
Fever	53 (32.9)
Cough	56 (34.8)
Fatigue	23 (14.3)
Time interval, Mean (SD)	
From arrival to symptom onset	2.4 (3.1)
From arrival to confirmed	2.7 (3.1)

## Discussion

During the COVID-19 pandemic, human-to-human transmission has been reported by many research groups, especially from asymptomatic cases to close contacts [14–17]. In this study, we reported 161 laboratory-confirmed cases from 4492 passengers with suspected COVID-19 infection, and by epidemiological investigation inferred whether these passengers might have been infected during air travel. Our findings suggest that the universal use of face masks on the flight, together with the airplane's ventilation system, likely prevented all secondary cases of COVID-19.

Previous studies reported that passengers on aircraft were prone to infection during epidemics of respiratory infectious diseases, including Severe Acute Respiratory Syndrome (SARS), Middle East Respiratory Syndrome (MERS), and influenza A (H1N1) [18–20]. In theory, in-flight transmission of COVID-19 could occur *via* direct physical contact, droplet spread, aerosol, and suspended small particles [21–23]. In this study, we found only two patients confirmed as infected who might have acquired the infection during the flight or the incubation period before boarding. The reasons for the low infection risk may be as follows. Firstly, almost every passenger and crew member used a face mask as a tool to stem transmission in cabins during air travel. Although some passengers would have had to remove their mask to eat and drink, we did not find any evidence that passengers might be infected in this regard. Secondly, the air circulation pattern on the aircraft is side to side, air enters the cabin from the top, circulates across the aircraft, and exits the cabin near the floor (Fig 1A) [24], a pattern which can effectively prevent respiratory infectious disease in cabins. Thirdly, control measures conducted at the airport played an important role. PEK designated T3-D as a special area for international aircraft docking, where all passengers suspected with COVID-19 infection were screened through quarantine inspection, laboratory testing for COVID-19, and isolation and medical observation for 14 days. These control measures were very effective for containment of COVID-19 [25–26].

Other studies on the clinical characteristics of COVID-19 have reported that most patients with confirmed infection presented with fever, cough, and fatigue [27–28]. However, in our study only 32.9% of confirmed patients had fever. Therefore, our findings indicate that temperature screening alone is not an effective way to contain the spread of COVID-19 at exit or entry ports for international flights, because infected individuals might be within the incubation period during which they do not express appropriate symptoms or are completely asymptomatic. Most of the confirmed cases were young women, the mean age of passengers was 28.6 years, and 26% of the cohort were students who were returning to China after the COVID-19 epidemic had begun to recede.

With the consideration of all these factors, we modified the non-steady Wells-Riley equation to better characterise the infectivity of COVID-19 during air travel. The estimated infectivity of COVID-19 was 375 quanta per hour, while the effective infectivity with face mask was only 4 quanta per hour. These results demonstrate two facts: firstly, the infection risk of COVID-19 could be much higher than H1N1(100 quanta per hour) and MERS-CoV(50 quanta per hour). And even though, the second fold reveals the almost negligible risk (4 quanta per hour) with face mask and well functioned ventilation system. The estimated infectivity and modified model can be utilised by individuals to evaluate the risk of air travel, and by governments or airlines to guide their operation policy.

## Conclusion

During the pandemic of COVID-19, our findings suggest that the risk of infection in aircraft was negligible. Universal use of face masks on the flight, together with the plane's ventilation system, likely prevented all secondary cases of COVID-19. Personal protection equipment on all flights should be strongly encouraged.

## Data Availability

https://doi.org/10.6084/m9.figshare.14332694.v1 [29]
